# Computational Investigation Identified Potential Chemical Scaffolds for Heparanase as Anticancer Therapeutics

**DOI:** 10.3390/ijms22105311

**Published:** 2021-05-18

**Authors:** Shraddha Parate, Vikas Kumar, Danishuddin, Jong Chan Hong, Keun Woo Lee

**Affiliations:** 1 Division of Applied Life Science, Plant Molecular Biology and Biotechnology Research Center (PMBBRC), Gyeongsang National University (GNU), 501 Jinju-daero, Jinju 52828, Korea; parateshraddha@gmail.com; 2 Division of Life Sciences, Department of Bio & Medical Big Data (BK4 Program), Research Institute of Natural Science (RINS), Gyeongsang National University (GNU), 501 Jinju-daero, Jinju 52828, Korea; vikaspathania777@gmail.com (V.K.); danish.info16@gmail.com (D.)

**Keywords:** Heparanase, pharmacophore modeling, virtual screening, molecular docking, molecular dynamics simulations, binding free energy calculations, MM/PBSA

## Abstract

Heparanase (Hpse) is an endo-β-D-glucuronidase capable of cleaving heparan sulfate side chains. Its upregulated expression is implicated in tumor growth, metastasis and angiogenesis, thus making it an attractive target in cancer therapeutics. Currently, a few small molecule inhibitors have been reported to inhibit Hpse, with promising oral administration and pharmacokinetic (PK) properties. In the present study, a ligand-based pharmacophore model was generated from a dataset of well-known active small molecule Hpse inhibitors which were observed to display favorable PK properties. The compounds from the InterBioScreen database of natural (69,034) and synthetic (195,469) molecules were first filtered for their drug-likeness and the pharmacophore model was used to screen the drug-like database. The compounds acquired from screening were subjected to molecular docking with Heparanase, where two molecules used in pharmacophore generation were used as reference. From the docking analysis, 33 compounds displayed higher docking scores than the reference and favorable interactions with the catalytic residues. Complex interactions were further evaluated by molecular dynamics simulations to assess their stability over a period of 50 ns. Furthermore, the binding free energies of the 33 compounds revealed 2 natural and 2 synthetic compounds, with better binding affinities than reference molecules, and were, therefore, deemed as hits. The hit compounds presented from this in silico investigation could act as potent Heparanase inhibitors and further serve as lead scaffolds to develop compounds targeting Heparanase upregulation in cancer.

## 1. Introduction

The extracellular matrix (ECM) plays a major role in providing a physical scaffold for all the tissues and also helps to maintain the biochemical processes important for tissue homeostasis [1]. The supramolecular proteins (collagen, laminin, elastin, fibronectin) interact with the ubiquitous macromolecules heparan sulfate proteoglycans (HSPGs) in the cell surface and ECM, to maintain the cellular framework [2]. Heparanase (Hpse) (EC 3.2.1.166) is a principal endo-β-D-glucuronidase that catalyzes the cleavage of glycosaminoglycan heparan sulfate (HS) side chains of HSPGs into smaller fragments of 10–20 sugar units, thus modulating the HS function [3]. The mammalian Hpse enzyme was isolated from the placenta and later from platelets and its activity contributes to disassembly and remodeling of ECM and basement membrane [2,4]. Notably, these events result in upregulation of cell migration, invasion and release of HS-bound angiogenesis factors [2]. Markedly upregulated Hpse levels trigger the MMP-9, hepatocyte growth factor (HGF) and vascular endothelial growth factor (VEGF) expression, thereby leading to cancer progression [5,6,7]. The upregulation of Hpse expression levels has also been reported in numerous solid and hematological malignancies, including ovarian, pancreatic, brain, bladder, prostate, colon, liver, breast, sarcoma and myeloma [8,9]. Moreover, Hpse overexpression is also linked with aggressiveness of a variety of tumor cell types, and as observed clinically, its expression is also associated with increased tumor size, tumor progression enhancement, metastasis and poor prognosis [10]. These actions lead to pathological ailments, such as tissue inflammation and angiogenesis, therefore making Hpse a druggable target for developing anticancer therapeutics.

The pharmacological inhibition of Hpse overexpression has been accomplished through some HS mimetics, including Muparfostat (PI-88), Roneparstat (SST0001), Pixatimod (PG545) and Necuparanib (M402), which have entered clinical trials as Hpse inhibitors [11,12]. PI-88 (Figure S1) is a combination of sulfated di- to hexasaccharides and it progressed to the Phase III of clinical trials for hepatocellular carcinoma. However, it demonstrated hematologic side effects when administered along with Docetaxel [13,14]. Roneparstat (Figure S1), with an IC_50_ value in the nanomolar range, is an N-acetylated glycol-split heparin and is in clinical trial for advanced multiple myeloma [15,16]. Pixatimod (Figure S1) is a fully sulfated hexasaccharide and a dual inhibitor of Hpse and angiogenesis, presently in Phase I of clinical trials for advanced solid tumors [17,18]. Necuparanib is a glycol-split HS mimetic and is currently in Phase-I/II clinical trials for metastatic pancreatic cancer when combined with gemcitabine and nab-paclitaxel [19,20]. Apart from the HS mimetic inhibitors, Zhao and co-workers identified a semisynthetic and highly sulfated oligosaccharide carbohydrate, JG3, from marine oligomannurarate [21]. JG3 inhibits both angiogenesis and metastasis and additionally blocks tumor growth. However, JG3 displays poor anticoagulant activity, and therefore, demonstrates low toxicity than other polyanionic compounds [1]. Another such marine-derived inhibitors for Hpse are a family of poly sulfated polygalactans originating from red algae and exhibiting anti-metastatic activity by inhibition of tumor-derived Hpse [22]. RK-682 (3-hexadecanoyl-5-hydroxymethyltetronic acid) (Figure S1), isolated from the mycelia of *Streptomyces* sp. 88–682, also displays an inhibitory activity for Hpse and the derivative 4-benzyl-RK-682 was also found to possess Hpse inhibitory activity (IC_50_ = 17 µM) [23,24]. Apart from the above-mentioned inhibitors, nucleic acid-based inhibitors such as Defibrotide have also been used to modulate the Hpse anti-cancer effect [1,25]. Defibrotide (Figure S1) is an orally bioavailable Hpse inhibitor, isolated from porcine intestinal mucosa, decreasing Hpse expression in multiple myeloma cell lines [26]. The developed Hpse inhibitors are predominantly carbohydrate-based compounds possessing heparin-like properties. However, these mimetics bind to heparin binding domains (HBD) flanking the Hpse active site, and therefore, are not specific for Hpse. Moreover, they interact with distinct heparin-binding proteins with off-target effects and unknown consequences [2]. Further disadvantages include their heterogeneous structures, which adds to their ambiguity as viable drugs for human use [2].

The discovery of small molecule Hpse inhibitors is desirable because of their efficient optimization for oral administration and promising pharmacokinetic properties, thereby resulting in an improved patient therapeutic compliance [27]. The polysulfonated naphthylurea-based small molecule, Suramin (Figure S1), inhibits melanoma Hpse and B16 melanoma cell invasion [28,29]. However, Suramin demonstrated adrenal insufficiency, neurotoxicity and renal toxicity along with anticoagulant-mediated blood dyscrasias, and therefore, failed to advance into clinical trials [1]. Additionally, several synthetic small molecules of various scaffolds have been reviewed in exclusive details by Mohan et al. [1], classifying them into benzazoles [27,30,31,32], thiazoles [33], oxazines [34,35,36,37,38,39,40], quinolines [41,42], glucans [41] and triazolo-thiadiazoles [2]. Apart from the inhibitors, Aspirin, which is a non-steroidal anti-inflammatory drug, was also found to inhibit Hpse by interacting with Glu225 in its catalytic site and observed to inhibit Hpse-mediated cancer cell migration, VEGF release and angiogenesis, both in vitro and in vivo [43].

Presently, a smaller quantity of small molecule inhibitors with promising pharmacokinetic properties are reported in literature for Hpse inhibition and the available HS mimetic inhibitors have failed at various stages of clinical trials. The search for new small molecule inhibitors with novel chemical scaffolds and the aforesaid perspectives prompted us to investigate natural as well as synthetic molecules as potential therapeutics targeted against Hpse. To attain this objective, we have carried out a ligand-based common-feature pharmacophore modeling study exploiting the shared chemical features of a few potent Hpse inhibitors, stated above. Accordingly, using the developed model as a query, we screened for compounds mapping our model, from a well-known InterBioScreen (IBS) database. We additionally checked their drug-likeness and performed molecular docking with the structure of Hpse. The acquired docked complexes were escalated further for evaluating their stability in physiological conditions. Subsequently, we subjected the simulated complexes to binding free energy calculations and confirmed two molecules each from natural and synthetic sources with better binding affinity than the reference compounds as hits.

## 2. Results

**Figure 1:**
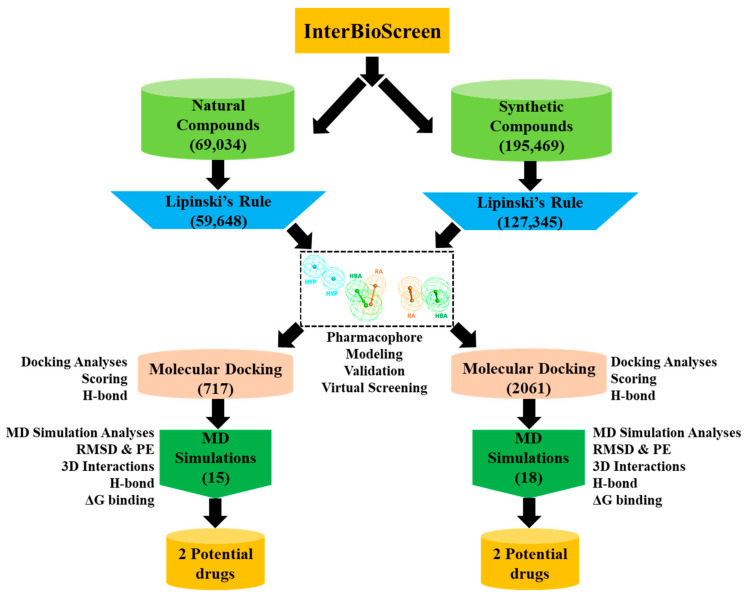
Flowchart depicting the working methodology in the current study used for the identification of potential Heparanase inhibitors.

In the present in silico investigation, a ligand-based pharmacophore modeling approach employing a series of computational techniques have been applied for the identification of potential Hpse inhibitors. The schematic representation of the study is summarized as below (Figure 1).

### 2.1. Common Feature Pharmacophore Model

**Figure 2:**
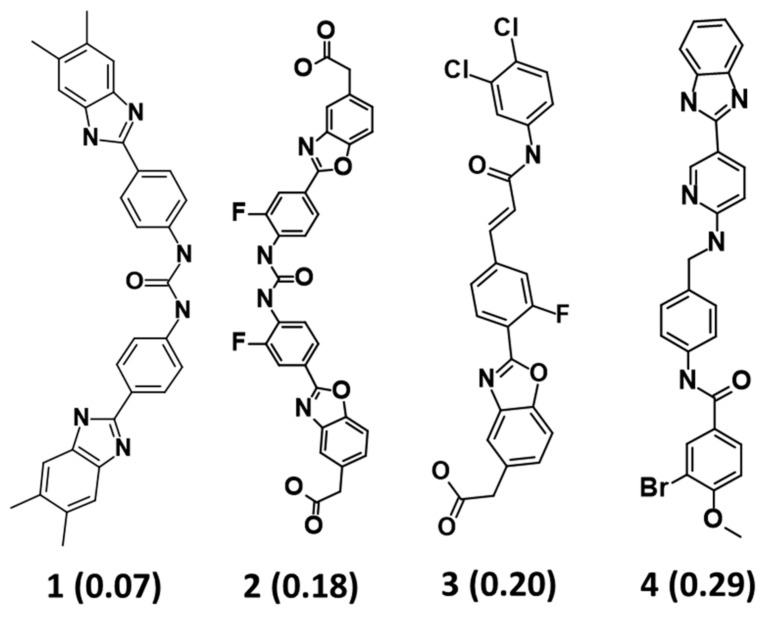
Two-dimensional (2D) structures of four active compounds used as training set for pharmacophore hypotheses generation. The inhibitory activity value (IC_50_) for each compound is shown in parentheses (µM).

**Figure 3:**
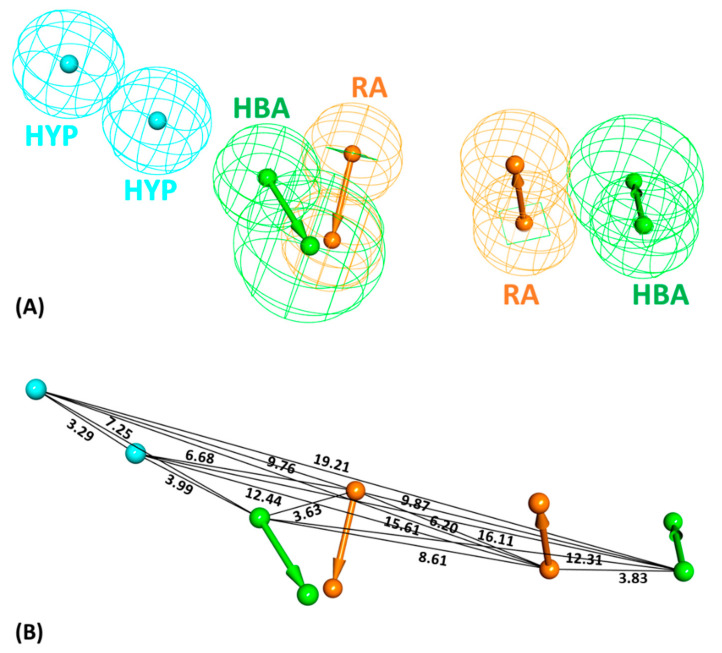
(**A**) The 3D representation of the selected pharmacophore model, Hypo1, containing two hydrophobic (HYP: cyan), two ring aromatic (RA: brown) and two hydrogen bond acceptor (HBA: green) features. (**B**) The interfeature distance (Å) among the features of Hypo1.

The *Feature Mapping* protocol availed prior to model generation revealed the crucial ring aromatic (RA), hydrogen bond acceptor (HBA) and hydrophobic (HYP) features from four structurally diverse and well-known Hpse inhibitors as a training set (Figure 2), required for Hpse inhibition. Accordingly, the *Common Feature Pharmacophore Generation* module using the *HipHop* algorithm resulted in 10 model hypotheses with 5 or 6 features each. The rank of the generated models ranged from 65.96–71.08 (Table 1). For the evaluation of the ranks, features and alignment of inhibitors with the generated hypotheses, Hypo1 with the highest rank 71.08 was selected as the most reliable pharmacophore model. The model selected from the above step encompasses two RA, two HBA and two HYP features (Figure 3). The chosen model, Hypo1 was escalated for further validation by the Güner-Henry (GH) approach.

### 2.2. Decoy Set Validation of the Pharmacophore Model

The selected model, Hypo1, was assessed for its robustness in retrieving active Hpse molecules from a mixed database of active and decoy compounds. This decoy set validation was initiated by the DS *Ligand Pharmacophore Mapping* module, which retrieved the four inhibitors used in pharmacophore generation from a given external database of 100 compounds (4 active + 96 decoys). Accordingly, the goodness of fit (GF) score was calculated as 0.72, which was found near the ideal model range value of 1 (Table 2) [44,45]. The value of the GF score confirmed that our model is robust for further predicting active and potent Hpse compounds from an external database reasonably well.

### 2.3. Drug-Likeness Evaluation and Virtual Screening of InterBioScreen Database

In the present in silico investigation, natural (69,034) and synthetic (195,469) compounds from the IBS database (Figure 1) were chosen for pharmacophore-based virtual screening. Prior to screening, a drug-like database of 186,993 compounds was prepared by Lipinski’s rule of five (Ro5) from the above natural and synthetic compounds. Subsequently, the validated Hypo1 was used as a query to screen this drug-like database, mapping a total of 2778 compounds with the pharmacophoric features. The drug-like compounds derived from this virtual screening strategy were escalated for molecular docking with the Hpse molecular structure.

### 2.4. Molecular Docking of Drug-Like Compounds with Heparanase

The process of molecular docking depends on the prediction of the binding mode and the interaction with the catalytic site of a protein [46]. A total of 2778 compounds procured from virtual screening were subjected to docking with the GS3 Hpse model structure. Preceding this docking process, the GOLD software authenticity was checked by re-docking the bound ligand, resulting in an acceptable root mean square deviation (RMSD) of 0.78 Å (Figure S2). Following the GOLD docking validation, the mapped compounds were docked with the Hpse molecular structure using the same coordinates utilized for bound ligand. Experimental studies revealed that molecules 1 and 2 (Figure 2) were reported to inhibit Hpse with the lowest inhibitory concentration. Therefore, to filter the true Hpse binding compounds, we considered both molecules as reference compounds and their docking scores as cutoff. Our docking analyses revealed that molecule 1 (REF1) displayed a Goldscore of 55.30 and a Chemscore of −27.79, while molecule 2 (REF2) demonstrated a Goldscore of 67.43 and a Chemscore of −24.35 (Table S1). Further using these scores as cutoff, 15 natural and 18 synthetic compounds with higher docking scores and favorable interactions with the active site residues (Asp62, Asn64, Thr97, Glu225, Asn227, Lys231, Gln270, Arg272, Glu343, Gly349, Gly350, Ala388 and Tyr391) were selected (Table S1). These interactions were characterized by numerous bonds including hydrogen, hydrophobic and van der Waals. Finally, the above 33 complexes obtained from docking were escalated to molecular dynamics simulations for evaluating their stability.

### 2.5. Molecular Dynamics Simulation Analysis

The docked complexes of the 33 compounds with Hpse were taken as initial coordinates to check their stability at the atomistic level via molecular dynamics (MD) simulations. In total, 33 systems were prepared and subjected to simulation production run of 50 ns each, along with 2 simulation systems for REF inhibitors. Finally, the resulting MD simulation systems were ranked according to their binding free energies (BFE) with Hpse, via Molecular Mechanics Poisson–Boltzmann Surface Area (MM/PBSA) methodology (Table S1). The MM/PBSA results revealed that REF1 and REF2 displayed with BFE value of −74.61 kJ/mol and −83.51 kJ/mol, respectively. Using the energy values of REF compounds as standard for the selection of potential inhibitors, we obtained four compounds with better BFE values, and therefore, these were considered as hit molecules (Table 3 and Table S2). Additionally, the stability of the selected hits was studied in terms of backbone RMSD, hydrogen bonds and potential energy plots. Furthermore, the binding interaction of the selected hits and REF compounds with the Hpse active site residues was scrutinized from the average structure taken from the last 5 ns of stable MD trajectories.

#### 2.5.1. Analysis of Stability and Binding Free Energy

**Figure 4:**
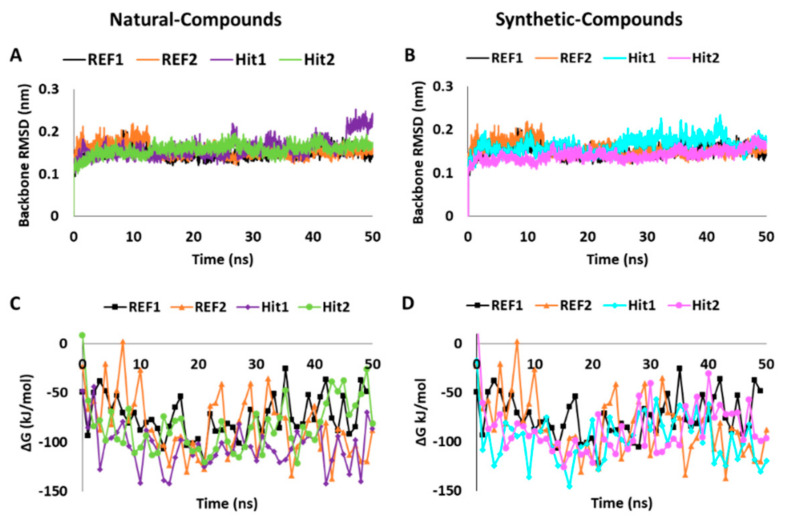
Molecular dynamics simulation analysis plots of Heparanase with the reference (REF) and Hits displaying (**A**,**B**) the backbone root mean square deviation (RMSD) and (**C**,**D**) the binding free energy (∆G_bind_) values. The left (**A**,**C**) and right (**B**,**D**) columns represent the analysis for natural and synthetic compound hits, respectively.

The backbone RMSD of simulated complexes was used to assess the stability and the systems were observed to be stable for the entire simulation period of 50 ns (Figure 4A,B). The hits acquired from the natural and synthetic IBS database presented with a convergent RMSD below 0.3 nm (Table 3). Concurrently, the BFE ∆G_bind_ values were calculated by generating 50 frames from the entire simulation run and plotted accordingly (Figure 4C,D). The hits from natural compounds Hit1 (STOCK1N-70463) and Hit2 (STOCK1N-48729) from the IBS database displayed a BFE value of −104.579 kJ/mol and −83.751 kJ/mol, respectively (Table 3 and Table S2, Figure 4C). Additionally, the hits from synthetic compounds Hit1 (STOCK1S-95244) and Hit2 (STOCK1S-71515) from the IBS database demonstrated a BFE value of −96.193 kJ/mol and −86.806 kJ/mol, respectively (Table 3 and Table S2, Figure 4D). The BFE values of our hits were observed to be comparably better than the BFE values of the REF compounds stated above. Hence, from this analysis, it can be perceived that our natural and synthetic hits have better affinity towards Hpse. Moreover, the entire energy of all systems was observed to remain stable as seen from the potential energy plots for all complexes (Figure S3A,B). In addition to the RMSD, BFE and potential energy, the analysis of hydrogen bonds over the 50 ns simulation period suggested that our hits demonstrate a higher number of bonds throughout the simulation run (Figure S3C,D, Table 3).

#### 2.5.2. Binding Mode and Molecular Interactions with Heparanase Active Site

The binding mode and interaction of our hits was scrutinized in detail by calculating the average structure from the last 5 ns of simulation run for each Hpse compound complex. 

The inhibitor REF1 was observed to display one hydrogen bond with catalytic residue Glu225. In addition, REF1 formed hydrophobic bonds with residues Tyr298 (π-π T-shaped), Val384 (alkyl) and Tyr391 (π-alkyl). REF1 also formed bonds with Thr60, Asp62, Gly95, Gly96, Thr97, Ser228, Arg272, His296, Glu343, Gln383 and Ala388, characterized by van der Waals interactions (Figure S4A,C, Table 4). The binding of inhibitor REF2 was rendered by hydrogen bonds with residue Gly349 and hydrophobic bonds with residues- Arg272 (π-cation) and Tyr391 (π-π T-shaped). The Hpse catalytic residues Thr97, Gln270, Pro271, Tyr348, Gly350, Gln383, Gly389 and Asn390 form van der Waals interactions with REF2 (Figure S4B,D, Table 4).

**Figure 5:**
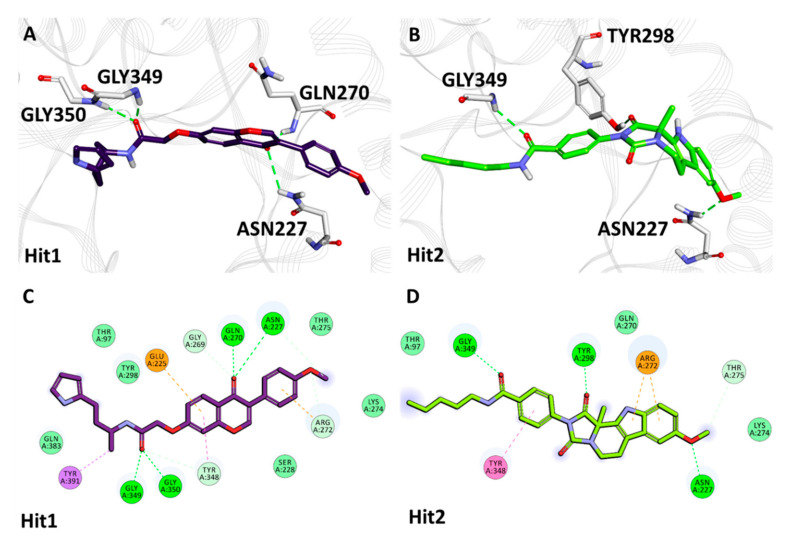
The 3D and 2D intermolecular interactions of natural compound hits (Hit1: **A**,**C**; Hit2: **B**,**D**) with the active site residues of Heparanase.

The average structure of Hit1, acquired from natural compounds, revealed that Hit1 demonstrated hydrogen bonds with Gln270, Asn227, Gly349 and Gly350 and hydrophobic interactions with Glu225 (π-anion), Arg272 (π-cation), Tyr348 (π-π T-shaped) and Tyr391 (π-sigma). Moreover, residues Thr97, Ser228, Lys274, Thr275, Tyr298 and Gln383 hold Hit1 in the Hpse catalytic pocket via van der Waals interactions (Figure 5A,C, Table 4). The identified Hit2 obtained from natural compounds was observed to interact with Asn227, Tyr298 and Gly349 via hydrogen bonds. Hit2 additionally formed bonds with Arg272 (π-cation) and Tyr348 (π-π T-shaped) via hydrophobic interactions. Residues Thr97, Gln270 and Lys274 hold Hit2 in the Hpse active site via van der Waals bonds (Figure 5B,D, Table 4).

**Figure 6:**
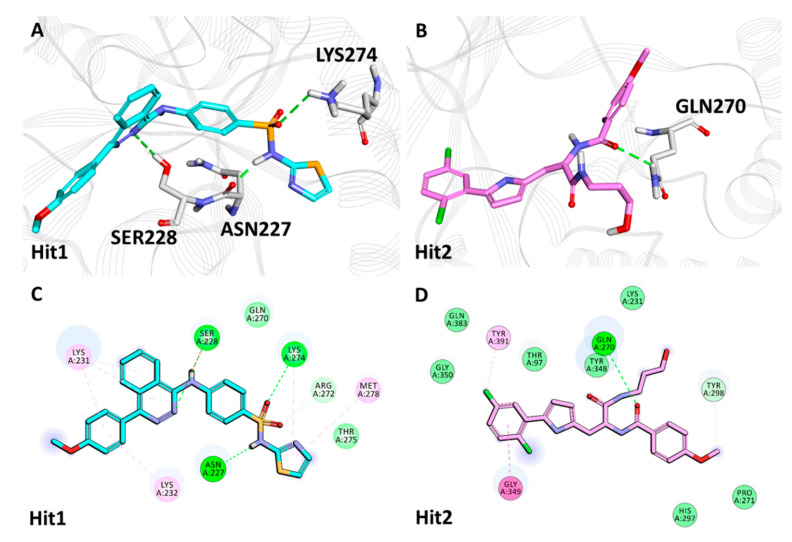
The 3D and 2D intermolecular interactions of synthetic compound hits (Hit1: **A**,**C**; Hit2: **B**,**D**) with the active site residues of Heparanase.

The representative structure of Hit1 attained from virtual screening of synthetic compounds displayed hydrogen bonds with Hpse catalytic residues Asn227, Ser228 and Lys274. Hit1, moreover, formed π-alkyl hydrophobic bonds with Lys231, Lys232, Lys274 and Met278 and van der Waals interactions with Gln270 and Thr275 (Figure 6A,C, Table 4). Furthermore, Hit2 obtained from synthetic compounds exhibited hydrogen bonds with Gln270 and hydrophobic bonds with Gly349 (amide π-stacked) and Tyr391 (π-alkyl). Additionally, Hit2 was supported by Thr97, Lys231, Pro271, His297, Tyr348, Gly350 and Gln383 via van der Waals interactions (Figure 6B,D, Table 4).

Overall, from the MD simulation analyses explained above, our identified hits display stability throughout the 50 ns of simulation run and also demonstrate comparably better binding affinities than REF inhibitors as observed from their BFE values. Moreover, the proposed hits in this study demonstrate intermolecular interactions with the key residues of Hpse catalytic site. Therefore, we anticipate that our hits can be deemed a good fit for Hpse inhibition.

## 3. Discussion

Human Hpse is an endoglucuronidase that cleaves HS side chains, an indispensable component of the ECM. This event leads to remodeling of the ECM, causing a release of growth factors and cytokines bound to HS. The release of growth factors further promotes pathological processes, including angiogenesis, migration of immune cells, inflammation and metastasis. Quintessentially, all cancers examined to date have been reported to upregulate Hpse activity, instigating tumor growth and metastasis with poor patient survival [47]. Therefore, Hpse has emerged as a valid druggable target for developing effective anti-cancer therapeutics. The advent of Hpse inhibitors has resulted in the development of carbohydrate-based molecules with heparin-mimicking properties [11,25,48]. These heparin mimics bind to heparin-binding domains flanking the Hpse catalytic site, thereby inhibiting HS cleavage. Four such heparin mimetics, Roneparstat, Necuparanib, Mupafostat and Pixatimod, are presently in clinical trials for different cancerous ailments. However, mimetic inhibitors result in off-target effects and are not Hpse-specific, causing them to bind with heparin-binding protein domains [2]. Such mimics, additionally, are heterogeneous in their structures (both in composition and chain length), further adding to their vagueness as viable Hpse drugs for human use. These effects limit their standardization, product characterization, biological data interpretation, and may also affect their delivery route [27]. The discovery and development of small molecule Hpse inhibitors is particularly desirable owing to their satisfactory pharmacokinetic properties and optimization, leading to oral administration. Although remarkable progress has been made in the development of small molecule Hpse inhibitors, no drugs able to modulate its activity has reached the clinical setting. Taking into account the aforementioned viewpoints, we pursued our research objective to identify both natural as well as synthetic small molecules as Hpse inhibitors by applying *Catalyst/HipHop*-based common-feature pharmacophore modeling.

The *Catalyst* pharmacophore modeling program queries interactions of compounds with the target protein on the basis of two hypothesis generation methods, including *HypoGen* and *HipHop* [49,50]. The *HypoGen* method illuminates the correlation between the chemical binding features of the compounds and their biological activities. The *HipHop* method, on the other hand, focuses on the chemical features which are common to the dataset of the most active compounds with a narrow activity range [51]. This hypothesis generation method produces pharmacophore models independent of the in vitro biological activity of the training set compounds [52,53]. Therefore, the objective of the present study was to acquire the active Hpse inhibitors with unique scaffolds encompassing acetic acid, benzamide, urea and benzazolyl derivatives for developing a pharmacophore model to inhibit the upregulation of Hpse (Figure 2).

The chemical features of the aforementioned active inhibitors were exploited by the *Catalyst/HipHop* program, which generated 10 pharmacophore hypotheses with distinct ranks and features (Table 1). Among the 10 generated models, the best 3D pharmacophore model with a ranking score of 71.08 was selected, composed of two ring aromatic, two hydrogen bond acceptor and two hydrophobic as quintessential features, required for Hpse inhibition (Figure 3). Our findings were also in accordance with a previously published study by Gozalbes et al., where a four-point pharmacophore model was developed consisting of hydrogen bond donor, acceptor and hydrophobic features as most essential for Hpse inhibition [42]. Gozalbes et al. and team successfully identified the anti-malarial drug, Amodiaquine displaying Hpse inhibitory activity employing their pharmacophore model [41]. Therefore, we argue that our pharmacophore model, generated with similar features as previous studies, gives reliable results for retrieving compounds that display a better binding affinity towards Hpse. The chosen Hpse pharmacophore model with the highest rank score was further validated by the decoy set validation method, generating a GF score of 0.72, which is near the ideal model range value of 1 (Table 2) [53,54,55]. The validated model was, thus, considered to be robust for retrieving molecules from an external database, and therefore, the resultant model was allowed to screen the InterBioScreen database composed of natural (69,034) and synthetic (195,469) compounds (Figure 1). Prior to virtual screening, the total number of molecules from both the subsets was reduced to 59,649 (natural) and 127,345 (synthetic) drug-like compounds via Lipinski’s Ro5 filtration. The screening of both the subgroups resulted in 717 (natural) and 2061 (synthetic) compounds, which were further subjected to molecular docking with the Hpse molecular structure. The crystal structure of human Hpse complexed with a tetrasaccharide inhibitor dp4 (PDB ID: 5E9C) [56] provided vital insights into the architecture of the Hpse binding cleft. Hpse is produced as a preproenzyme, and after proteolytic activation, two distinct subunits, including the N-terminal 8 kDa (residues Gln36-Glu109) and C-terminal 50 kDa (residues Lys158-Ile543), produced a mature form of Hpse [4,27,57]. The catalytic site of Hpse is characterized by a narrow channel, with active site residues, Glu225 (proton donor) and Glu343 (proton acceptor) placed in the middle of the channel [58]. The inhibitor binding site is characterized on one side by the heparin binding domain 2 (HBD2; residues 270–280) and on the other side by heparin binding domain 1 (HBD1; residues 158–171) accommodating the terminal iduronic acid of inhibitor dp4 in PDB ID: 5E9C. The model of Hpse built recently by Madia et al. was utilized for our molecular docking studies to devise a putative binding mode for our drug-like compounds with Hpse [27]. This model was built by adding the connecting GS3 ((Gly-Ser) × 3) peptide using MODELER 9.16 software, in which the 8 kDa and 50 kDa subunits are connected, and is, therefore, referred to as the catalytically active form of Hpse. Molecular docking with Hpse resulted in 15 (natural) and 18 (synthetic) compounds demonstrating higher Goldscores and lower Chemscores than the reference inhibitors (Table S1). The above 33 compounds also demonstrated interactions with the key residues of the Hpse binding pocket. Therefore, the acquired 33 compounds were evaluated in physiological conditions by molecular dynamics simulations, and their binding free energies were computed by MM/PBSA calculations (Table S1). The calculated energies were compared with the energy values of the reference inhibitors. REF1 and REF2 displayed BFE values of −74.612 kJ/mol and −83.519 kJ/mol, respectively (Table 3 and Table S1). The MM/PBSA calculations revealed better BFE values for two of our identified hits with Hit1 and Hit2 from natural compounds, demonstrating −104.579 kJ/mol and −83.751 kJ/mol, respectively (Table 3 and Table S2). The BFE calculations for hits obtained from synthetic compounds revealed Hit1 and Hit2, with considerably better values of −96.193 kJ/mol and −86.806 kJ/mol, respectively (Table 3 and Table S2). The BFE scores by MM/PBSA enable the entropic distribution of the total ∆G_bind_ energy into identifiable contributions. These individual contributions are characterized by van der Waals, electrostatic, polar solvation and SASA energy. As observed from the BFE distribution analysis, the van der Waals and electrostatic forces provided the maximum driving force for binding of our hits with Hpse. Additionally, the contribution of van der Waals interaction in binding of Hit1 from natural (−169.280 ± 18.050 kJ/mol) as well as synthetic (−163.420 ± 16.897 kJ/mol) sources was observed to be near the contributing range of van der Waals interaction in binding of REF2 (−173.780 ± 16.684 kJ/mol) (Table S2). The entropic distribution of the total BFE suggests that our hits also contribute comparably better in terms of SASA energy and electrostatic energy (Table S2).

The hits achieved from natural and synthetic compounds were further scrutinized for their molecular interactions with Hpse catalytic site residues. Literature reviews on the residues targeting Hpse via hydrogen bonds revealed Glu225, Asn227, Lys231, Gln270, Arg272, Lys274, Glu343, Gly349, Gly350 and Ala388 as vital for Hpse inhibition [27,57,58,59]. Accordingly, our obtained hits were observed to target Gln270, Asn227, Lys274, Gly349 and Gly350 via hydrogen bonds (Table 4, Figure 5A,B and Figure 6A,B). Further assessment of the literature revealed that the previously reported inhibitors with effective biological activity against Hpse targeted residues Ser163, Glu225, Asn227, Lys231, Gln270, Arg272, Lys274, His296, Tyr298, Glu343, Tyr348, Gly349, Gly350, Gly351, Glu383, Ala388, Gly389 and Tyr391 via hydrophobic and/or van der Waals interactions [23,27,57,58,59]. Consequently, our hits also demonstrated interactions with the above residues characterized by van der Waals- and π-mediated bonds (Table 4, Figure 5C,D and Figure 6C,D). As perceived from the hydrogen bond analysis plots, our hits demonstrated a higher number of bonds than the reference inhibitors (Figure S3C,D). Moreover, the hits were found to be stable throughout the 50 ns of simulation, as seen from their RMSD (Figure 4A,B) and potential energy plots (Figure S3A,B). The Hpse-hit complexes were also scrutinized at 0 ns and 50 ns to observe the difference in their interactions with Hpse active site residues (Figure S5). It was perceived that our hits formed higher number of van der Waals interactions at 0 ns, while most of these interactions were lost at the end of 50 ns. Additionally, the superimposed complex structures revealed a slight deviation in the binding pose of Hit1 from the synthetic source at 50 ns. Despite the pose deviancy, it was noticed that Hit1 retained similar interactions with the catalytic site residues of Hpse (Figure S5C). Correspondingly, the alignment of identified hits with Hypo1 indicated that our hits portray the pharmacophoric features reasonably well (Figure S6).

Additionally, to further confirm the toxicity properties of our final hit compounds, the *Toxicity Prediction (TOPKAT)* module implanted in DS was utilized to evaluate three properties. The TOPKAT module depends on the notion of Quantitative Structure-Toxicity Relationship (QSTR) models and computes toxicity properties which include rodent carcinogenicity, AMES mutagenicity and skin irritancy. According to the U.S. National Toxicology Program (NTP), the compound’s rodent carcinogenicity property is evaluated by testing it in both sexes of mouse. The TOPKAT results demonstrated our hits to be non-carcinogenic in both sexes of mouse models. Furthermore, our hits were also observed to be non-AMES mutagenic and non-skin irritant (Table 5). Investigation of oral administration and pharmacokinetic (PK) properties of final hit compounds is essential to avoid their failure in clinical trials. Therefore, the PK properties of our identified hits as well as those of REF inhibitors were calculated and compared by the online tool *pkCSM* (Table 6) (accessed on 12 May 2021, http://biosig.unimelb.edu.au/pkcsm/) [60]. Given an input molecule, *pkCSM* predicts PK properties using graph-based signatures. Accordingly, *pkCSM* predicts various properties encompassing molecular weight, rotatable bonds, water solubility, intestinal absorption, BBB permeability, CYP2D6 inhibitory activity, hERG inhibitory prediction, total clearance and renal OCT2 substrate prediction. The *pkCSM* results demonstrated that our hits display a moderate level of water solubility, thus confirming that they have good oral bioavailability. Moreover, Caco-2 cell lines are widely employed as an in vitro model in pre-clinical investigations for predicting the likely gastrointestinal permeability of drugs [61]. It was observed that Hit2 from a natural source and Hit1 from a synthetic source displayed better Caco-2 permeability than the REF inhibitors, while Hit1 from a natural source and Hit2 from a synthetic source demonstrated a permeability closer to the acceptable range. A literature survey revealed that molecules with an intestinal absorption (IA) level of <30% are classified as being poorly soluble. Intriguingly, all our identified hits displayed IA levels >30%, similar to that observed for the potent Hpse REF inhibitors. Additionally, the skin permeability property of a given compound of interest is considered for the development of transdermal drug delivery, and it was observed that our hits demonstrated acceptable skin permeability scores (>−2.5). The P-glycoprotein (P-gp) is an extensively studied ATP-binding cassette (ABC) transporter regulating the uptake and efflux of drugs, thereby helping in their absorption [62]. In the present study, our identified hits were observed to be P-gp substrates similar to REF1, except for Hit1 from a synthetic source and REF2. Moreover, all of our hits were predicted to be inhibitors of P-gp. Furthermore, our hit compounds displayed low BBB permeability, thereby limiting the chances of nervous system-related toxicity. The metabolic performance of our hits and REF inhibitors was also assessed by CYP2D6 isoform of cytochrome P450 inhibition. The *pkCSM* results predicted that our hits were observed to be non-inhibitors of CYP2D6 similar to REF inhibitors, and thus, can be metabolized in the liver. The total clearance (TC) parameter for the excretion of drugs exhibited that Hit1 from a natural source demonstrates an acceptable TC value similar to REF1, while other hits displayed low TC values similar to that observed for REF2. In addition, our hits were not found to be substrates of renal organic cation transporter 2 (OCT2), which is an essential factor to be considered for the renal clearance of drugs. This illustrated that our hits do not have the potential for adverse interactions with co-administered OCT2 inhibitors. Additionally, hERG I is an essential determinant of normal repolarization of cardiac action potential, and its inhibition leads to cardiotoxicity. The *pkCSM* results predicted that our hits do not inhibit hERG I. Finally, it was perceived that even though REF inhibitors demonstrate good inhibitory activities against Hpse, they do not obey all the Lipinski’s Ro5 rules. Both REF1 and REF2 display a molecular weight of more than 500 Da and lipophilicity (LogP) on the higher level. Compared to REF inhibitors, our hits obeyed all the rules, except for Hit1 from a synthetic source, which displayed LogP slightly greater than the acceptable limit. The above overall properties and the low molecular weights of our hit compounds plays an essential role in their oral absorption. At this stage, note that there are numerous servers for predicting PK properties and the results of different servers are not always the same.

In addition to the aforementioned analysis, the identified natural and synthetic hits were searched in the PubChem chemistry database (accessed on 15 April 2021, https://pubchem.ncbi.nlm.nih.gov/) [63] by entering their SMILES (simplified molecular-input line-entry system) IDs [64] to check if our hits have been evaluated in the literature, against Hpse. From the PubChem analysis, it was observed that our hits have not been assessed against Hpse before, and hence, can be considered as valuable therapeutics against Hpse-mediated ailments. Moreover, from the IUPAC name of our hits, it can be perceived that Hit2 from both synthetic and natural compounds represent compounds from benzamide origin (Table 7) and similar molecules of this source have been explored and reported before by Xu et al. [31]. On the other hand, Hit1 from both natural and synthetic compounds indicate molecules of acetamide and sulfonamide origin, respectively (Table 7). The small molecules from the aforementioned origins have not been reported in the literature yet. Overall, we anticipate that our hits may be effective drug candidates as potent therapeutics and can be recommended for further evaluation against Hpse.

The laboratory in vitro research necessitates chemicals and other techniques, which is a time-consuming and tedious process [65]. Therefore, we accessed the experiment-free prediction method for assessing the inhibitory behavior of our hit compounds. Deep learning models have progressed recently to predict the inhibitory activity of the compounds. PaccMann (accessed on 12 May 2021, https://ibm.biz/paccmann-aas) is one such web-based drug sensitivity platform designed to utilize multimodal attention-based neural networks [66]. Moreover, PaccMann is an effective validation toolbox used for drug repurposing approaches and has an R^2^ value of 0.86 along with an RMSE (root mean square error) value of 0.89, highlighting the strong correlations between the resultant data generated by the server and the experimentally estimated values. Accordingly, the SMILES IDs of our hit compounds and REF inhibitors were supplied to the platform as an input and the sensitivity against cancer cell lines was predicted in terms of their IC_50_ values. The IC_50_ values were estimated for the ovarian (A2780), lung (A549) and breast (MCF-7) cancer cell lines, as Hpse is overexpressed in the aforementioned particular cancers. Additionally, the IC_50_ values were also predicted for Ewing’s sarcoma (SK-ES-1), multiple myeloma (MM1S) and hepatocellular carcinoma (HepG2) cell lines, owing to Hpse dysregulation in the cancer subtypes. The prediction of IC_50_ values for all hits was observed to be low or in the similar range as that for REF inhibitors in all cancer cell lines, except for Hit2 from a synthetic source, which was observed to predict higher IC_50_ values (Table S3). A similar study was also recently performed by Thirunavukkarasu et al. [67], who utilized the PaccMann server to successfully predict the anticancer sensitivity on 77 lung cancer cell lines.

## 4. Materials and Methods

### 4.1. Dataset Preparation and Pharmacophore Model Generation

A dataset of four well-known Hpse inhibitors, as reported in the literature [1], composed of distinct scaffolds and different maximal inhibitory concentration (IC_50_) values, was chosen as the training set for the generation of pharmacophore model. These four inhibitors consisted of an acetic acid (IC_50_ = 0.2 µM) [33], benzamide (IC_50_ = 0.29 µM) [31], urea (IC_50_ = 0.075 µM) [30] and symmetrical benzazolyl (IC_50_ = 0.18 µM) [57] derivatives endowed with Hpse inhibitory activity. Accordingly, the 3D structures of the chosen compounds were downloaded from BindingDB [68], manually checked and energy minimized employing the *Minimize Ligands* module in Discovery Studio (DS) v.18 (Accelrys, San Diego, CA, USA). Prior to model generation, the *Feature Mapping* protocol in DS was employed for identifying the common chemical features in the training set compounds. The features predicted from the above-mentioned step were used as inputs for the generation of a model using the *Common Feature Pharmacophore Generation* tool of DS. This ligand-based pharmacophore approach utilizes the *HipHop* algorithm to extract features common to a set of limited active molecules [49]. The *BEST/Flexible* conformation generation, along with an energy threshold of 20 kcal/mol and interfeature distance of 2.97 Å, was used to produce a maximum of 255 conformations. A total of 10 hypotheses are generated with parameters, including the comprised features, hypothesis rank, direct hit, partial hit and maximum fit values.

### 4.2. Validation of the Generated Model

Pharmacophore validation is a crucial step in assessing the pharmacophore robustness for retrieving active compounds from a given dataset. The best pharmacophore hypothesis generated by *HipHop* was validated by the Güner-Henry (GH) scoring method, also known as the decoy set method [69]. The GH validation was instigated by subjecting the generated pharmacophore model to an external dataset (D) of 100 compounds with four active (A) molecules used in pharmacophore generation. The selected pharmacophore hypothesis was used as a 3D query employing *Ligand Pharmacophore Mapping* protocol of DS for acquiring the goodness of fit (GF) score in the range of 0 (null model) and 1 (ideal model) [55]. The equation mentioned below was utilized for calculating the GF score value, where Ht signifies the total number of hits retrieved by the pharmacophore model and Ha denotes the active molecules retrieved in Ht:

$$GF=\left(\frac{Ha}{4HtA}\right)\;\left(3A+Ht\right)\times \;\left\{1-,\frac{Ht-Ha}{D-A}\right\}$$

### 4.3. Drug-Like Database Generation and Virtual Screening of InterBioScreen Database

The virtual screening strategy in this study used the validated pharmacophore as a query to search the InterBioScreen (IBS) database composed of natural compounds (69,034) as well as synthetic compounds (195,469). Prior to screening, the compounds were filtered for their drug-like attributes on the basis of Lipinski’s Rule of Five (Ro5) and Veber’s rule by employing the *Filter by Lipinski and Veber Rules* module of DS. Ro5 signifies that the potential drug-like compound exhibits a molecular weight of 500 Da, an octanol/water partition coefficient (log P) of less than 5, less than 5 hydrogen bond donors and 10 hydrogen bond acceptors [70]. Additionally, Veber’s rule of less than 10 rotatable bonds was applied to acquire drug-like compounds for further analysis [71]. The validated pharmacophore model was subsequently used to screen these drug-like databases by engaging the *Ligand Pharmacophore Mapping* protocol of DS using the *FAST/Flexible* fitting method. The mapped drug-like compounds were consequently chosen for molecular docking with Hpse.

### 4.4. Molecular Docking of Screened Drug-Like Compounds with Hpse

Docking studies were initiated by adopting the model of human GS3 Hpse, previously developed by Madia et al. from the PDB ID: 5E9C (resolution: 1.73 Å) (accessed on 24 November 2020, www.rcsb.org) [56], where Hpse is complexed with heparin tetrasaccharide inhibitor dp4 [27]. The mature human Hpse (UniProtKB ID: Q9Y251) is a heterodimer structure encompassing two chains—N-terminal 8 kDa (residues Gln36-Glu109) and C-terminal 50 kDa (residues Lys158-Ile543)—noncovalently assembled into a (β/α)_8_-TIM barrel fold [57]. The developed model of Hpse was built in a way that the 8 kDa and 50 kDa chains are connected by linker peptide GS3 ((Gly-Ser) × 3) and represents the catalytically active form of human Hpse. The binding site in the Hpse model was defined as a sphere of 16 Å by using the *Define and Edit Binding Site* tool of DS with X, Y and Z co-ordinates of −22.86, 13.98 and 59.98, respectively. Prior to the docking process, the Genetic Optimization for Ligand Docking (*GOLD* v5.2.2) [72] protocol was validated by re-docking the co-crystallized benzazolyl inhibitor of the aforementioned Hpse model. The drug-like natural and synthetic compounds obtained from virtual screening of IBS database were minimized and prepared by employing the *Minimize Ligands* DS protocol. Consequently, the compounds acquired were docked with the Hpse model by allowing for generation of 10 conformers per ligand. The obtained conformations were clustered to achieve the largest cluster, from which the compounds were evaluated on the basis of two scoring criterions [73]—Goldscore (high) and Chemscore (low) [74]—as well as the molecular interactions with the Hpse catalytic site residues (Asp62, Asn64, Thr97, Glu225, Asn227, Lys231, Gln270, Arg272, Glu343, Gly349, Gly350, Ala388 and Tyr391).

### 4.5. Molecular Dynamics Simulation of Identified Natural and Synthetic Compounds

Molecular dynamics (MD) simulations are applied to understand the protein-ligand interactions at the atomic level in order to scrutinize their conformational flexibility and structural stability under physiological conditions [75,76]. The complexes obtained from the process of molecular docking were subjected to MD simulations in GROningen MAchine for Chemical Simulations (*GROMACS* v2018) [77] with the docked structures of compounds with Hpse as initial coordinates. The protein and the compounds were parametrized by CHARMm27 [78] and SwissParam [79] fast force field generation tools, respectively. All simulation systems were immersed in a dodecahedron water box of TIP3P solvent model and neutralized by the addition of 16 Cl^-^ ions. Prior to system equilibration, energy minimization of each simulation system was performed by the steepest descent algorithm in order to avoid steric clashes [80]. Systems were further subjected to two-stage equilibration process composed of NVT (constant number of particles, volume and temperature) and NPT (constant number of particles, pressure and temperature) for 500 ps each. The NVT ensemble uses a V-rescale thermostat [81] to equilibrate the system temperature at 300 K, while the NPT ensemble uses the Parrinello-Rahman barostat [82] to maintain the system pressure at 1.0 bar. Systems equilibrated by the above steps were subjected to MD simulation run of 50 ns each under periodic boundary conditions. Long-range electrostatic interactions were calculated with Particle Mesh Ewald (PME) [83] approach with a cutoff radius of 10 Å and the LINCS algorithm [84] restrained the bond lengths of heavy atoms. The MD outcomes were further visualized and scrutinized in DS and visual molecular dynamics (VMD) [85].

### 4.6. Binding Free Energy Calculations

Calculation of the binding affinity of small molecule inhibitors with their target proteins represents a quintessential role to prioritize compounds before their experimental evaluation [86]. To evaluate the binding affinities of our compounds with Hpse, the Molecular Mechanics Poisson-Boltzmann Surface Area (MM/PBSA) in GROMACS was utilized by implementing the ‘*g_mmpbsa*’ tool [87]. For this purpose, 50 snapshots were selected evenly for the entire simulation run of 50 ns and the binding free energy ∆G_bind_ was computed as:

$$\Delta G_{bind}=\;G_{complex}\left(G_{protein}+\;G_{ligand}\right)$$

## 5. Conclusions

A ligand-based common-feature pharmacophore model exploiting the features shared by a set of active inhibitors revealed essential criteria required for Heparanase inhibition. The model composed of six pharmacophoric features was validated and subsequently used as a query to screen drug-like compounds from the InterBioScreen database. A total of 2778 drug-like compounds acquired from pharmacophore mapping were assessed by molecular docking with Heparanase to gain insight on their binding. The 33 obtained compounds from docking analysis exhibited higher docking scores than the reference inhibitors and were able to inhibit Heparanase heterodimer by interacting with key active site residues. The acquired compounds were further evaluated in physiological conditions via molecular dynamics simulations and their binding affinities with Heparanase were computed by MM/PBSA calculations. Analysis of binding affinity revealed two hit compounds each from natural and synthetic databases displaying higher binding affinity than reference inhibitors for Heparanase inhibition. Additionally, the intermolecular interaction analysis revealed that the selected hits interact with key catalytic residues via hydrogen bonds, thus providing support in their selection as hit molecules. Furthermore, compounds of acetamide and sulfonamide scaffolds have not been previously reported as Hpse inhibitors. Therefore, compounds comprising aforementioned scaffolds can be considered as a novel source for future identification of Heparanase inhibitors. We anticipate that the identified scaffolds of hit compounds can be considered for drug optimization against Heparanase in the future.

## Figures and Tables

**Table 1:** Composition of the common-feature pharmacophore hypotheses generated by *HipHop* algorithm.

Sr. No.	Features ^a^	Rank ^b^	Direct Hit ^c^	Partial Hit ^d^	Max Fit ^e^
1	RA, RA, HBA, HBA, HYP, HYP	71.08	1111	0000	6
2	RA, RA, HBA, HBA, HYP, HYP	70.28	1111	0000	6
3	RA, RA, HBA, HBA, HYP, HYP	70.28	1111	0000	6
4	RA, RA, HBA, HBA, HYP, HYP	69.48	1111	0000	6
5	RA, HBA, HYP, HYP, HYPA	66.18	1111	0000	5
6	HBA, HYP, HYP, HYPA, HYPA	66.09	1111	0000	5
7	RA, HBA, HYP, HYP, HYPA	66.04	1111	0000	5
8	RA, HBA, HYP, HYP, HYPA	66.00	1111	0000	5
9	RA, RA, HBA, HYP, HYP	65.96	1111	0000	5
10	RA, RA, HBA, HYP, HYP	65.96	1111	0000	5

^a^ Features: RA: ring aromatic; HBA: hydrogen bond acceptor; HYP: hydrophobic; HYPA: hydrophobic aromatic. ^b^ Rank: The best hypothesis demonstrates the highest rank. The higher the rank score, the lower the likelihood of chance correlation. ^c^ Direct Hit: Value (1) signifies that the training set molecules mapped well to all chemical features of the hypothesis. ^d^ Partial Hit: Value (0) signifies that there was no partial mapping of the training set molecules with the hypothesis. ^e^ Max Fit: The maximum number of features in the hypothesis.

**Table 2:** Decoy set validation of Hypo1 from an external database composed of active and inactive Heparanase inhibitors.

Sr. No.	Parameters	Values
1	Total number of compounds in the database (D)	100
2	Total number of active compounds in the database (A)	4
3	Total number of hits retrieved by pharmacophore model from the database (Ht)	6
4	Total number of active compounds in the hit list (Ha)	4
5	% Yield of active ((Ha/Ht) × 100)	66.66%
6	% Ratio of actives ((Ha/A) × 100)	100%
7	False negatives (A-Ha)	0
8	False positives (Ht-Ha)	2
9	Goodness of fit score (GF)	0.72

**Table 3:** Molecular docking and molecular dynamics simulation analyses for reference (REF) inhibitors and selected potential hits from InterBioScreen (IBS) database against Heparanase.

Ligands (IBS ID/REF No.)	Docking Scores		MD Analyses		
	Goldscore	Chemscore	RMSD (Backbone)	Hydrogen Bond (Å)	Binding Free Energy (kJ/mol)
**Natural Compound Hits**					
Hit1 (STOCK1N-70463)	68.95	−32.00	0.16	2.16	−104.579 ± 20.649
Hit2 (STOCK1N-48729)	67.79	−30.66	0.15	0.98	−83.751 ± 26.469
**Synthetic Compound Hits**					
Hit1 (STOCK1S-95244)	74.92	−30.70	0.16	0.37	−96.193 ± 23.866
Hit2 (STOCK1S-71515)	67.53	−33.38	0.14	1.17	−86.806 ± 26.536
**Reference Inhibitors**					
REF1	55.30	−24.35	0.14	0.25	−74.612 ± 20.900
REF2	67.43	−24.35	0.15	1.13	−83.519 ± 31.504

**Table 4:** Molecular interactions of the compounds (reference and hits) with Heparanase active site residues obtained from stable molecular dynamics simulation trajectories.

Complex Name		Hydrogen Bond Interactions				van der Waals Interactions	π-π/π-alkyl Interactions
		Amino Acid	Amino Acid Atom	Ligand Atom	Distance (<3.05 Å)		
**Natural Compound Hits**							
Heparanase + Natural Compounds	**Hit1**	Asn227	HD22	O13	3.02	Thr97, Ser228, Gly269, Arg272, Lys274, Thr275, Tyr298, Tyr348, Gln383	Glu225, Tyr391
		Gln270	HN	O13	2.08		
		Gly349	HN	O18	1.90		
		Gly350	HN	O18	2.29		
	**Hit2**	Asn227	HD21	O29	2.07	Thr97, Gln270, Lys274, Thr275, Gly350	Arg272, Tyr348
		Tyr298	HH	O16	1.80		
		Gly349	HN	O19	2.61		
**Synthetic Compound Hits**							
Heparanase + Synthetic Compounds	**Hit1**	Asn227	O	H35	1.85	Gln270, Arg272, Thr275	Lys231, Lys232, Met278
		Ser228	HG	N6	2.68		
		Lys274	HZ2	O15	2.68		
	**Hit2**	Gln270	HE21	O15	2.60	Thr97, Lys231, Pro271, His297, Tyr298, Tyr348, Gly350, Gln383	Gly349, Tyr391
**Reference (REF) Inhibitors**							
Heparanase + Reference Inhibitors	**REF1**	Glu225	OE2	H66	1.74	Thr60, Asp62, Gly95, Gly96, Thr97, Ser228, Arg272, His296, Glu343, Gln383, Ala388	Tyr298, Val384, Tyr391
	**REF2**	Gln349	HN	O23	2.39	Thr97, Gln270, Pro271, Tyr348, Gly350, Gln383, Gly389, Asn390	Arg272, Tyr391

**Table 5:** Toxicity properties of identified natural and synthetic compound hits generated by *TOPKAT*.

Hits (IBS ^a^ ID)	Mouse Female Carcinogenicity	Mouse Male Carcinogenicity	AMES ^b^ Mutagenicity	Skin Irritancy
**Natural Compound Hits**				
Hit1 (STOCK1N-70463)	Non-Carcinogen	Non-Carcinogen	Non-Mutagen	Non-Irritant
Hit2 (STOCK1N-48729)	Non-Carcinogen	Non-Carcinogen	Non-Mutagen	Non-Irritant
**Synthetic Compound Hits**				
Hit1 (STOCK1S-95244)	Non-Carcinogen	Non-Carcinogen	Non-Mutagen	Non-Irritant
Hit2 (STOCK1S-71515)	Non-Carcinogen	Non-Carcinogen	Non-Mutagen	Non-Irritant

^a^ IBS: InterBioScreen; ^b^ AMES: Salmonella typhimurium reverse mutation assay.

**Table 6:** In silico assessment of pharmacokinetic (PK) properties for reference (REF) inhibitors and identified hits generated by *pkCSM*.

PK Properties	Natural Compound Hits		Synthetic Compound Hits		Reference Inhibitors		Cut-Off
	Hit1 (STOCK1N-70463)	Hit2 (STOCK1N-48729)	Hit1 (STOCK1S-95244)	Hit2 (STOCK1S-71515)	REF1	REF2	
Molecular weight	447.48	474.56	489.58	489.35	500.60	598.51	≤500 Da
LogP	4.57	4.33	5.30	4.53	7.65	6.47	<5
Rotatable Bonds	9	7	7	9	4	8	<10
HBA	6	4	8	5	3	7	≤10
HBD	1	2	2	3	4	4	≤5
Water solubility	−5.585	−5.078	−3.182	−4.998	−2.892	−2.905	<−10 insoluble to <0 highly soluble
Caco-2 permeability	0.585	1.169	1.101	0.564	0.754	−0.526	>0.90
IA (human)	94.26	100	97.45	82.54	100	64.80	>30
Skin permeability	−2.688	−2.802	−2.735	−2.752	−2.735	−2.735	>−2.5
P-gp substrate	Yes	Yes	No	Yes	Yes	No	No
P-gp I inhibitor	Yes	Yes	Yes	Yes	No	No	No
BBB permeability	−0.862	−0.854	−0.633	−1.136	−0.941	−2.352	>0.3 high to <−1 poor
CYP2D6 inhibitor	No	No	No	No	No	No	No
hERG I inhibitor	No	No	No	No	Yes	No	No
Total clearance	0.544	0.152	−0.023	−0.124	0.813	−0.171	<0.3 low to >0.7 high
Renal OCT2 substrate	No	No	No	No	Yes	No	No

Abbreviations—HBA: Hydrogen Bond Acceptor, HBD: Hydrogen Bond Donor, IA: Intestinal Absorption, P-gp: P-glycoprotein, BBB: Blood–Brain Barrier, hERG: human ether-a-go-go-related gene, OCT2: Organic Cation Transporter 2.

**Table 7:** Molecular structures and IUPAC names of identified hits from InterBioScreen database.

Compound Name	IUPAC Name	Molecular Structure
**Natural Compound Hits**		
Hit1	N-(4-(furan-2-yl)butan-2-yl)-2-((3-(4-methoxyphenyl)-4-oxo-4H-chromen-7-yl)oxy)acetamide	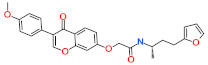
Hit2	(S)-4-(8-methoxy-11b-methyl-1,3-dioxo-5,6-dihydro-1H-imidazo[1 ‘,5′:1,2]pyrido[3,4-b]indol-2(3H,11H,11bH)-yl)-N-pentylbenzamide	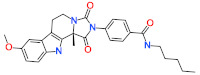
**Synthetic Compound Hits**		
Hit1	4-((4-(4-methoxyphenyl)phthalazin-1-yl)amino)-N-(thiazol-2-yl)benzenesulfonamide	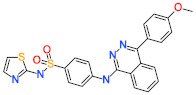
Hit2	(E)-N-(1-(5-(2,5-dichlorophenyl)furan-2-yl)-3-((3-hydroxypropyl)amino)-3-oxoprop-1-en-2-yl)-4-methoxybenzamide	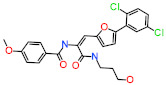

## Data Availability

Data are contained within the article.

## Supplementary Materials

The following are available online at https://www.mdpi.com/article/10.3390/ijms22105311/s1, Figure S1. Chemical structures of carbohydrate-based, nucleic acid-based and small molecule Heparanase inhibitors. Figure S2. Overlay of the docked pose (orange) of bound ligand with its GS3 Heparanase model conformation (mauve). Figure S3. Molecular dynamics simulation analysis plots of Heparanase with the reference (REF) and Hits displaying (A and B) potential energy and (C and D) hydrogen bonds. The left (A and C) and right (B and D) columns represent analysis for natural and synthetic compound hits, respectively. Figure S4. The 3D and 2D intermolecular interactions of reference (REF) compounds (REF1: A and C; REF2: B and D) with the active site residues of Heparanase. Figure S5. The superimposed complex structures and 2D intermolecular interactions of natural and synthetic compound hits with the catalytic residues of Heparanase at 0 ns and 50 ns. Figure S6. Alignment of identified hits with the pharmacophoric features of Hypo1. Subsections A and B represent Hit1 and Hit2 from natural compounds, respectively, whereas subsections C and D denote Hit1 and Hit2 from synthetic compounds of InterBioScreen database, respectively. Table S1. The docking and binding free energy scores of drug-like natural and synthetic compounds from the InterBioScreen (IBS) database with Heparanase. Table S2. The entropic distribution of the total binding free energy scores for reference (REF) inhibitors and selected potential hits from the InterBioScreen (IBS) database against Heparanase. Table S3. Assessment of anti-cancer drug sensitivity prediction for reference (REF) inhibitors and identified hits generated by PaccMann.
