# Supplementary material for: Computational Investigation Identified Potential Chemical Scaffolds for Heparanase as Anticancer Therapeutics
Source: Int J Mol Sci. 2021 May 18;22(10):5311. doi: 10.3390/ijms22105311 (PMC8157885; doi:10.3390/ijms22105311)
Supplement: Supplementary file 1 [file ijms-22-05311-s001.zip › ijms-1208042-supplementary.pdf]

International Journal of
Molecular Sciences

MDPI

Article

Computational Investigation Identified Potential Chemical Scaffolds for Heparanase as Anticancer Therapeutics

Shraddha Parate [1,†], Vikas Kumar [2,†], Danishuddin [2], Jong Chan Hong [1,*] and Keun Woo Lee [2,*]

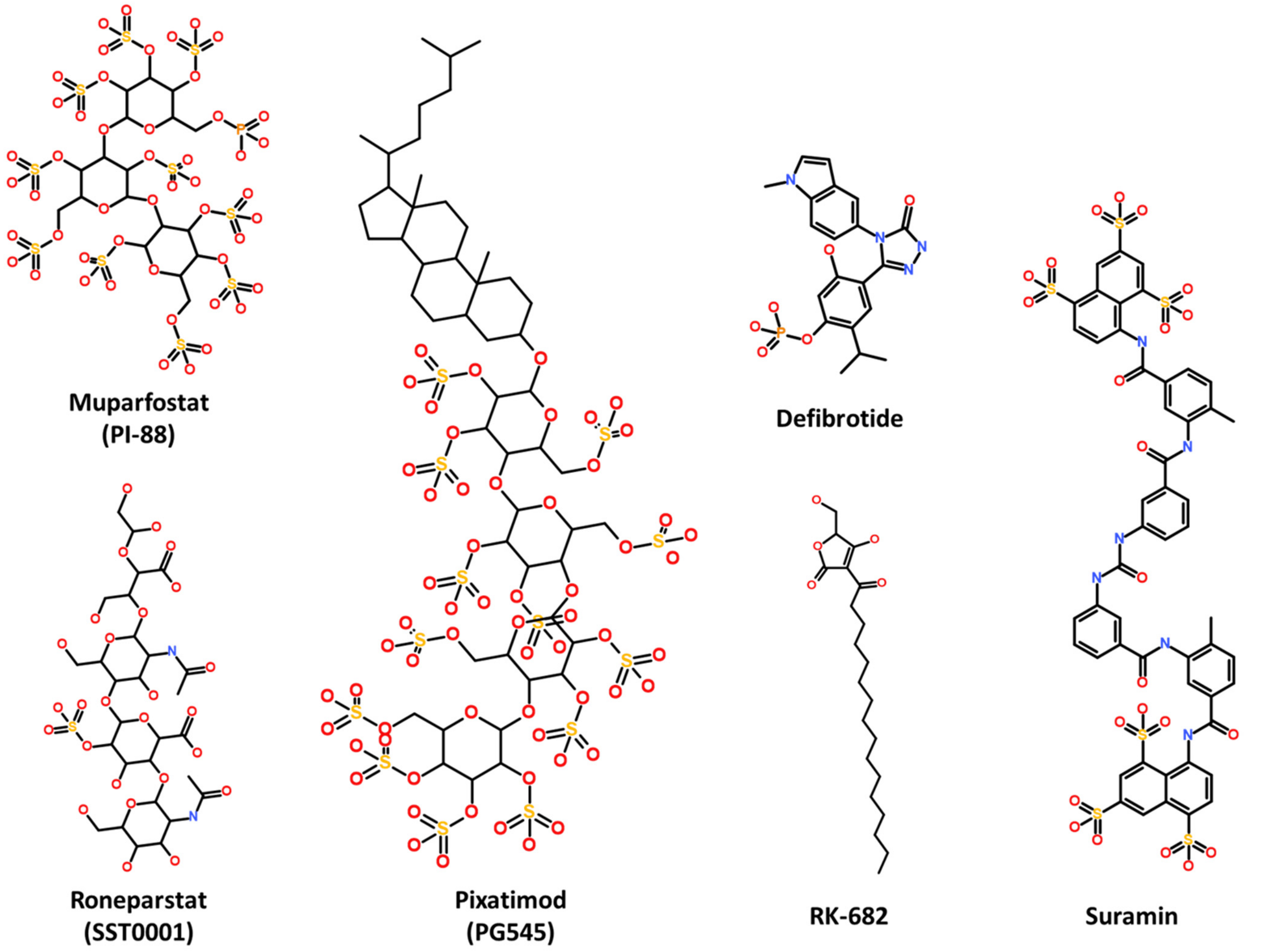

Figure S1. Chemical structures of carbohydrate-based, nucleic acid-based and small molecule Heparanase inhibitors.

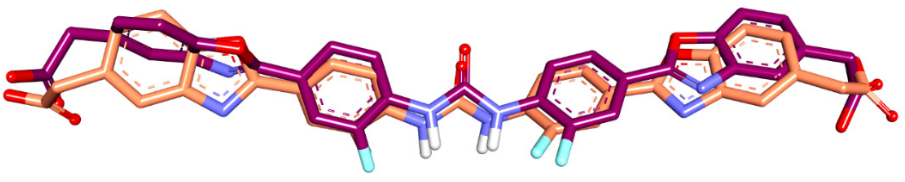

Figure S2. Overlay of the docked pose (orange) of bound ligand with its GS3 Heparanase model conformation (mauve).

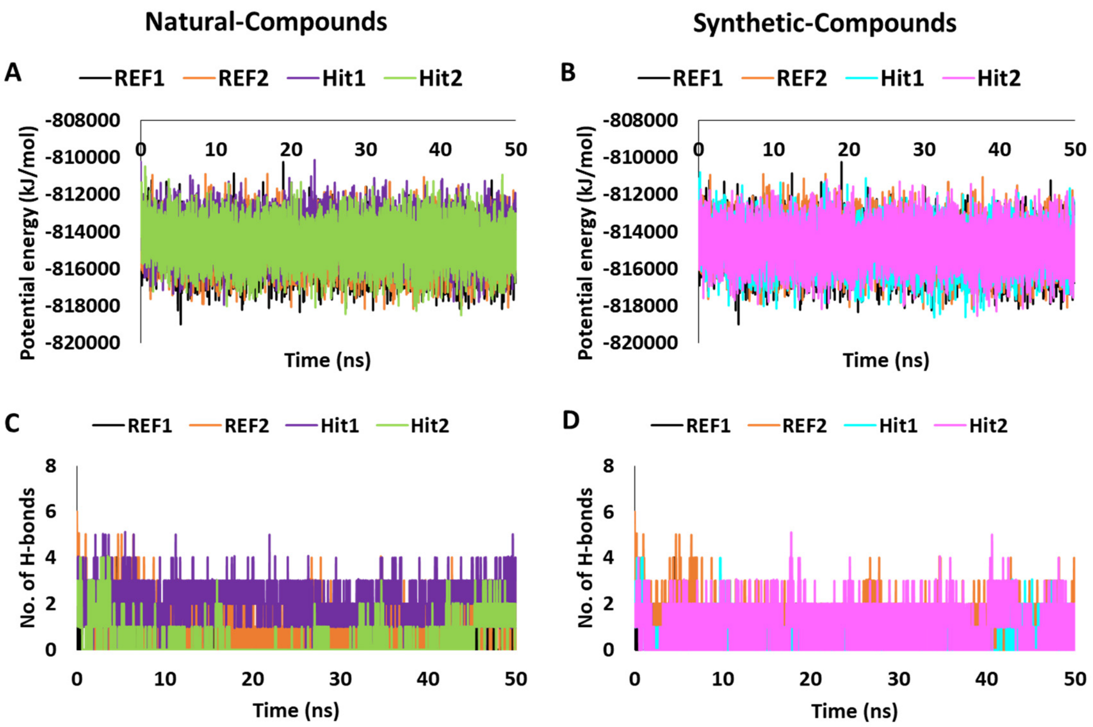

Figure S3. Molecular dynamics simulation analyses plots of Heparanase with the reference (REF) and Hits displaying (A and B) potential energy and (C and D) hydrogen bonds. The left (A and C) and right (B and D) columns represent analysis for natural and synthetic compound hits, respectively.

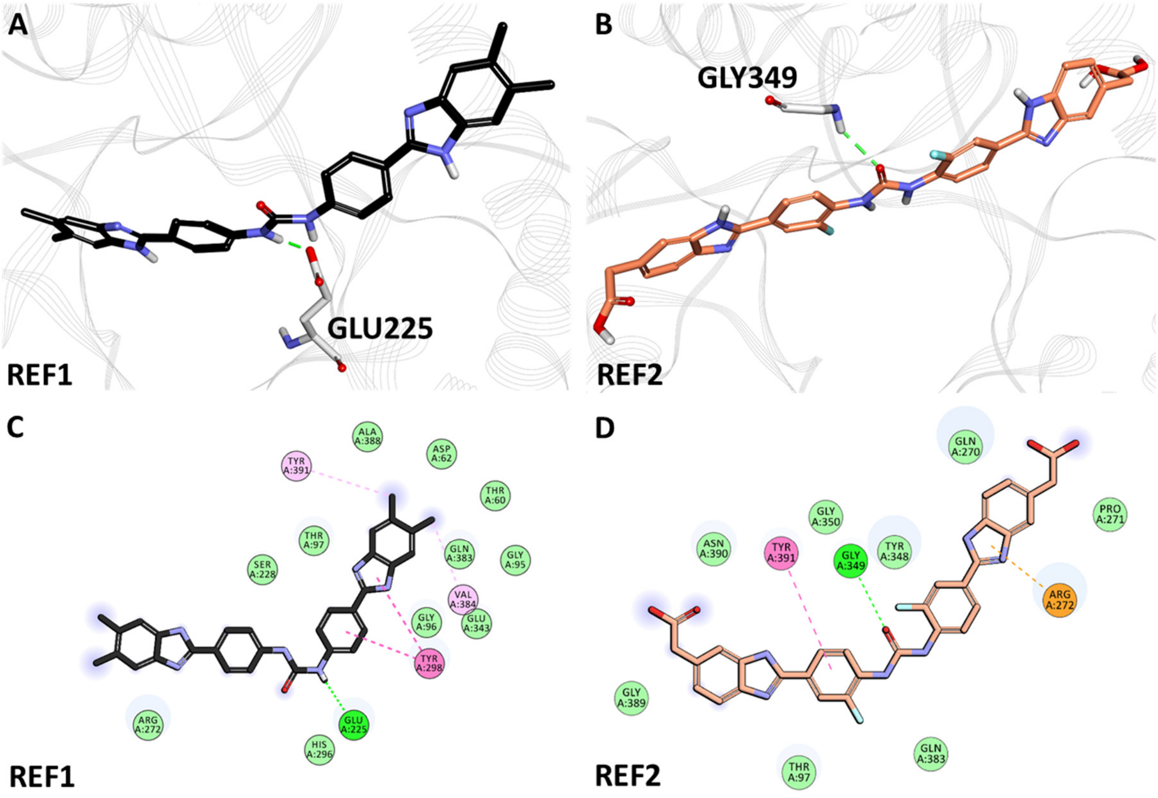

Figure S4. The 3D and 2D intermolecular interactions of reference (REF) compounds (REF1: A and C; REF2: B and D) with the active site residues of Heparanase.

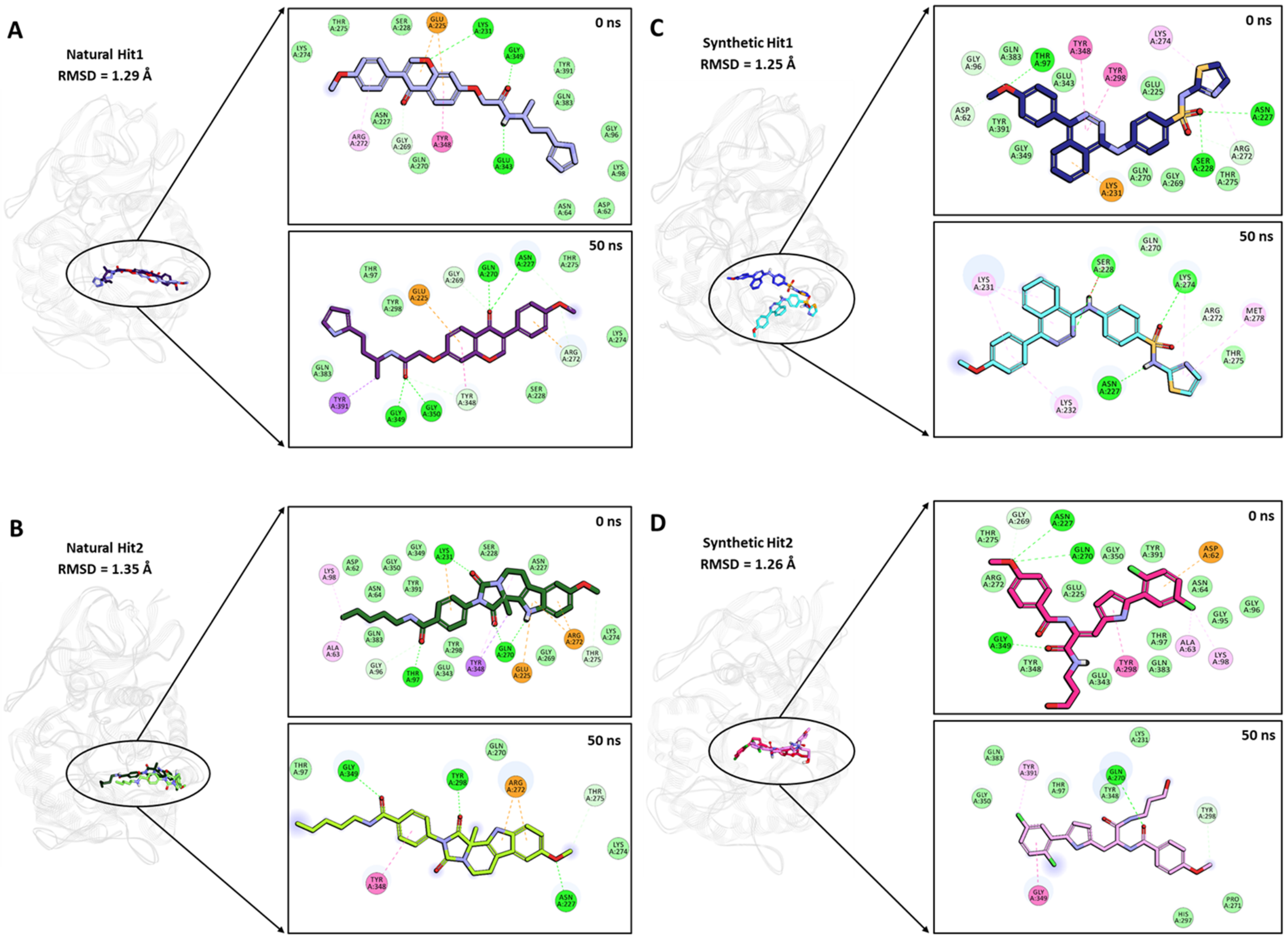

Figure S5. The superimposed complex structures and 2D intermolecular interactions of natural and synthetic compound hits with the catalytic residues of Heparanase at 0 ns and 50 ns.

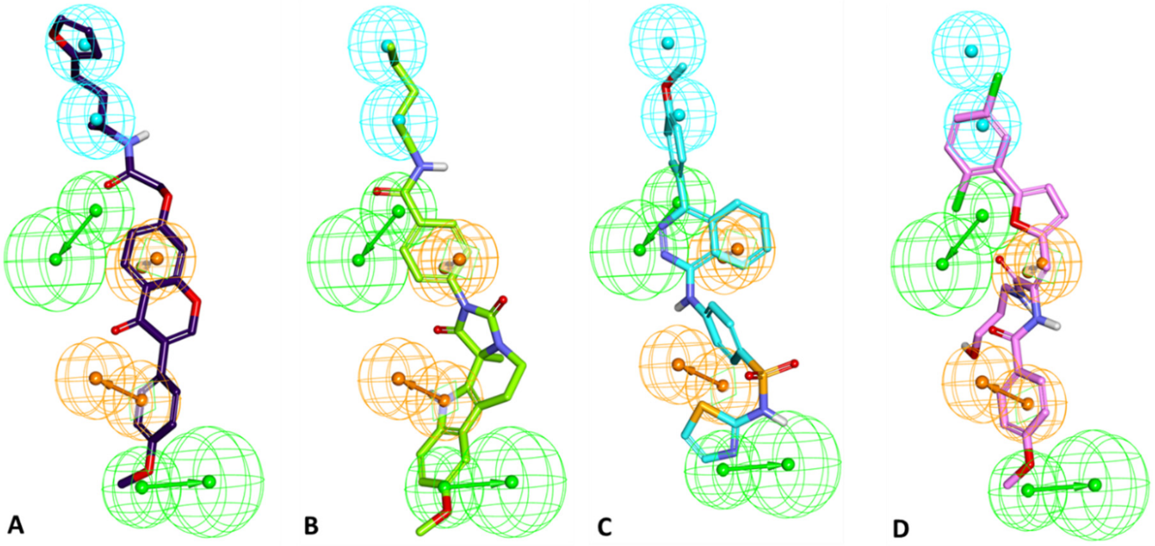

Figure S6. Alignment of identified hits with the pharmacophoric features of Hypo1. Subsections A and B represent Hit1 and Hit2 from natural compounds, whereas subsections C and D denote Hit1 and Hit2 from synthetic compounds of InterBioScreen database, respectively.

Table S1. The docking and binding free energy scores of drug-like natural and synthetic compounds from the InterBioScreen (IBS) database with Heparanase.

IBS ID/REF No.	SMILES ID	Docking Scores		Binding Free Energy Scores
		Goldscore	Chemscore	ΔG_{bind} (kJ/mol)
	**Natural Drug-Like Compounds**			
**STOCK1N-75678**	COC1=C(C=CC=C1)C(=O)NC2=CC=C(C=C2)C(=O)C3=CC4=C(CCN3C)C=C5OCOC5=C4OC	72.62	-32.54	-67.45
**STOCK1N-90022**	COC1=C(OC)C=C(C=C1)C2=COC3=CC(=CC=C3C2=O)OCC(=O)NC4=CC5=C(OCCO5)C=C4	70.95	-30.26	-44.12
**STOCK1N-71247**	COC1=CC=C(C=C1)C2=COC3=CC(=CC=C3C2=O)OCC(=O)NCCC4=C[NH]C5=C4C=CC=C5	70.67	-31.84	-54.42
**STOCK1N-21853**	COC1=CC2=C([NH]C3=C2CCNC3C4=CC=C(OC)C(=C4)COC5=CC=C(C=C5)[N](=O)=O)C=C1	69.62	-30.54	-39.03
**STOCK1N-59679**	COC1=CC=C(OC)C(=C1)C2=CC3=C(OC2=O)C=C(OC(=O)C4=CC(=CC(=C4)OC)OC)C=C3	69.31	-33.91	-59.23
**STOCK1N-45143**	COC(=O)C1=CC=C(COC2=CC3=C(C=C2)C(=O)\C(O3)=C\C4=CC=C(C=C4)C(=O)OC)C=C1	69.20	-34.19	-45.53
**STOCK1N-71407**	COC1=CC=C2OC(=O)C(=CC2=C1)C3=CC=C(OCC(=O)NC(C)CC4=CC=CO4)C=C3	69.03	-34.03	-68.02
**STOCK1N-70463**	COC1=CC=C(C=C1)C2=COC3=CC(=CC=C3C2=O)OCC(=O)NC(C)CCC4=CC=CO4	68.95	-32.00	-104.57
**STOCK1N-57934**	CCCOC(=O)COC1=CC=C2C=C(C(=O)OC2=C1)C3=CC(=O)OC4=C3C=CC(=C4)OC	68.95	-31.80	-51.10
**STOCK1N-22579**	COC1=CC2=C([NH]C3=C2CCNC3C4=CC=C(OC)C(=C4)COC5=CC=C(NC(C)=O)C=C5)C=C1	68.94	-34.87	-46.94
**STOCK1N-90307**	CC(=O)C1=CC(=CC=C1)NC(=O)COC2=CC=C3C(=O)C(=C(C)OC3=C2)C4=CC=CC=C4	67.98	-30.17	-55.66
**STOCK1N-48729**	CCCCCNC(=O)C1=CC=C(C=C1)N2C(=O)N3CCC4=C([NH]C5=C4C=C(OC)C=C5)C3(C)C2=O	67.79	-30.66	-83.75
**STOCK1N-67945**	CCCCNC(=O)C(C)OC1=CC2=C(C=C1)[N]3C(=C4C(OC)C(=CC=C4C5=NC=CC2=C35)OC)O	67.77	-43.38	-55.81
**STOCK1N-36801**	COC1=CC=C(C=C1OC)C(=O)OC2=CC=C3C(=O)C(=C(C)OC3=C2)C4=CC(=C(OC)C=C4)OC	67.61	-29.05	-61.54
**STOCK1N-68238**	COC1C(=CC=C2C3=NC=CC4=C3[N](C(=C12)O)C5=C4C=C(OCC(=O)NCC6=CC=CC=C6)C=C5)OC	67.53	-42.25	-67.13
	**Synthetic Drug-Like Compounds**			
**STOCK2S-58608**	CC1=CC=C(C=C1)C2=CC=C(OCC(=O)NC3=CC=C(C=C3)[S](=O)(=O)NC4=NC=CS4)C=C2	81.01	-30.43	-53.29
**STOCK3S-51955**	CC(C)CC1=CC=C(C=C1)C2=CSC(=N2)\C(=C\NC3=CC=C(C=C3)[S](=O)(=O)NC(N)=O)C#N	77.85	-32.04	-59.03
**STOCK2S-50669**	COC1=CC(=CC=C1OCC2=CC=C(C=C2)C(O)=O)[CH]=[N+]=[NH+][S](=O)(=O)C3=CC=C4C=CC=CC4=C3	77.73	-34.28	-71.35
**STOCK1S-47127**	COC1=CC(=C(C=C1)C(=O)\C=C\NC2=CC=C(C=C2)[S](=O)(=O)NC3=NC(=CC(=N3)C)C)OC	76.38	-31.12	-73.59
**STOCK3S-52245**	CCCOC1=CC=C(\C=C\C(=O)NC2=CC=C(C=C2)[S](=O)(=O)NC3=NC(=CC(=N3)C)C)C=C1	75.76	-34.62	-68.04
**STOCK3S-49803**	CCCOC1=CC=C(\C=C\C(=O)NC2=CC=C(C=C2)[S](=O)(=O)NC3=CC(=NC(=N3)C)C)C=C1	75.17	-34.60	-61.53

**STOCK1S-95244**	COC1=CC=C(C=C1)C2=NN=C(NC3=CC=C(C=C3)[S](=O)(=O)NC4=NC=CS4)C5=C2C=CC=C5	74.92	-30.70	-96.19
**STOCK2S-58772**	CCOC1=C(OCC)C=C(C=C1)C(=O)NC2=CC=C(C=C2)[S](=O)(=O)NC3=NC(=CC=N3)C	74.53	-31.32	-70.27
**STOCK2S-58619**	COC1=C(OC)C=C(C=C1)C(=O)NC2=CC=C(C=C2)[S](=O)(=O)NC3=NC(=CC(=N3)C)C	74.29	-31.00	-67.59
**STOCK2S-04448**	CCN(CC)C(=O)C1=CC=C(C=C1)C2=C3C=CC=CC3=C(NC4=CC=C(C=C4)[S](N)(=O)=O)N=N2	73.30	-36.28	-52.70
**STOCK3S-42907**	CC1=CC=C(C=C1)C2=N[N+](=C([O-])C3=C2C=CC=C3)CC(=O)NC4=CC=C(C=C4)C(=O)N5CCCCC5	72.48	-36.75	-55.61
**STOCK3S-57053**	CCCOC1=CC=C(C=C1)C(=O)NC2=CC=C(NC(=O)C3=CC4=C(O3)C=CC=C4)C(=C2)OC	71.47	-35.32	-73.41
**STOCK3S-43653**	COC1=CC=C(C=C1)C(=O)NC2=CC=C(CC3=CC=C(NC(=O)C4=CC=C(OC)C=C4)C(=C3)O)C=C2O	69.98	-31.57	-52.49
**STOCK3S-30539**	CCOC1=CC=C(C=C1)C2=C3C=CC=CC3=C(NC4=CC=C(C=C4)[S](N)(=O)=O)N=N2	69.29	-32.09	-59.31
**STOCK2S-94267**	CC[N]1C(=NN=C1C2=CC=C(C)C=C2)SCC(=O)[NH+]=[N+]=[C](C)C3=CC=C(OC(C)=O)C=C3	69.05	-31.47	-54.27
**STOCK3S-72317**	COC1=CC=C(NC(=O)C2CC(=O)N(CC3=CC4=C(OCO4)C=C3)C(S2)=NC5=CC=CC=C5)C=C1	67.85	-31.47	-67.31
**STOCK3S-52762**	CCC(=O)NC1=CC=C(C=C1)C2=NN=C(SCC(=O)C3=CC=C4C=CC=CC4=C3)[N]2C	67.80	-32.05	-54.64
**STOCK1S-71515**	COC1=CC=C(C=C1)C(=O)N\C(=C\C2=CC=C(O2)C3=C(Cl)C=CC(=C3)Cl)C(=O)NCCCO	67.53	-33.38	-86.80
**REF2**	OC(=O)CC1=CC2=C(OC(=N2)C3=CC=C(NC(=O)NC4=CC=C(C=C4F)C5=NC6=C(O5)C=CC(=C6)CC(O)=O)C(=C3)F)C=C1	67.43	-24.35	-83.51
**REF1**	CC1=CC2=C(C=C1C)N=C([NH]2)C3=CC=C(NC(=O)NC4=CC=C(C=C4)C5=NC6=C([NH]5)C=C(C)C(=C6)C)C=C3	55.30	-27.79	-74.61

Table S2. The entropic distribution of the total binding free energy scores for reference (REF) inhibitors and selected potential hits from InterBioScreen (IBS) database against Heparanase.

Ligands (IBS ID/REF No.)	van der Waals (kJ/mol)	Electrostatic (kJ/mol)	Polar solvation (kJ/mol)	SASA energy (kJ/mol)	Binding free energy ΔG_{bind} (kJ/mol)
**Natural Compound Hits**					
Hit1 (STOCK1N-70463)	-169.280 +/- 18.050	-86.216 +/- 15.175	171.068 +/- 26.205	-20.150 +/- 1.812	-104.579 +/- 20.649
Hit2 (STOCK1N-48729)	-147.049 +/- 24.361	-21.267 +/- 19.495	103.627 +/- 44.940	-19.062 +/- 2.438	-83.751 +/- 26.469
**Synthetic Compound Hits**					
Hit1 (STOCK1S-95244)	-163.420 +/- 16.897	-35.954 +/- 14.820	121.494 +/- 29.609	-18.313 +/- 1.647	-96.193 +/- 23.866
Hit2 (STOCK1S-71515)	-133.337 +/- 16.571	-44.788 +/- 20.534	107.469 +/- 45.892	-16.150 +/- 2.029	-86.806 +/- 26.536
**Reference Inhibitors**					
REF1	-135.600 +/- 13.852	-77.120 +/- 17.869	155.337 +/- 37.648	-17.230 +/- 2.079	-74.612 +/- 20.900
REF2	-173.780 +/- 16.684	-71.717 +/- 31.611	182.920 +/- 59.166	-20.942 +/- 2.278	-83.519 +/- 31.504

Table S3. Assessment of anti-cancer drug sensitivity prediction for reference (REF) inhibitors and identified hits generated by PaccMann.

Cancer Types	Natural Compound Hits (IC50)(μM)		Synthetic Compound Hits (IC50)(μM)		Reference Inhibitors (IC50)(μM)		Cell Lines
	Hit1 (STOCK1N-70463)	Hit2 (STOCK1N-48729)	Hit1 (STOCK1S-95244)	Hit2 (STOCK1S-71515)	REF1	REF2	
Ovarian cancer	0.528	0.556	0.522	0.599	0.562	0.569	A2780
Lung cancer	0.564	0.581	0.573	0.624	0.604	0.592	A549
Breast cancer	0.582	0.549	0.598	0.633	0.614	0.601	MCF-7
Ewing's sarcoma	0.548	0.567	0.557	0.611	0.593	0.583	SK-ES-1
Multiple myeloma	0.539	0.562	0.547	0.608	0.580	0.584	MM1S
Hepatocellular carcinoma	0.534	0.523	0.518	0.611	0.541	0.526	HepG2
